# Survival nomograms for colorectal carcinoma patients with lung metastasis and lung-only metastasis, based on the SEER database and a single-center external validation cohort

**DOI:** 10.1186/s12876-022-02547-9

**Published:** 2022-11-05

**Authors:** Lin-Lin Liu, Jun-Die Sun, Zuo-Lin Xiang

**Affiliations:** 1 Department of Radiation Oncology, Shanghai East Hospital, School of Medicine, Tongji University, Shanghai, 200120 China; 2 Department of Radiation Oncology, Shanghai East Hospital Ji’an hospital, Ji’an City, 343000 Jiangxi Province China

**Keywords:** Colorectal carcinoma, Lung metastasis, Prognosis, Nomogram, SEER database

## Abstract

**Background:**

We analysed the survival of colorectal cancer (CRC) patients with lung metastasis and lung-only metastasis and determined the risk factors for lung metastasis in CRC patients.

**Methods:**

Data from colorectal cancer patients with lung metastasis diagnosed from 2010 to 2015 were obtained from the SEER database. Survival was analysed using the Kaplan–Meier method and log-rank test, the Cox proportional hazards regression model, and a competing risk model. The predictive ability of the nomgram was assessed by the concordance index (C-index) and calibration curves. The data from the SEER database for the period 2016–2019 was used as an external validation set. The characteristics of 70 CRC patients treated at Shanghai East Hospital between 2016 and 2019 were retrospectively analysed and data from China was chosen as an external validation set.

**Results:**

The median survival time for colorectal cancer patients with lung metastasis was 12 months, while this value was 24 months in patients with lung-only metastasis. Among all CRC patients with lung metastasis, age, grade, T stage, N stage, presence of liver, brain or bone metastasis, anatomic site and surgery were related to overall survival (OS). In CRC patients with lung-only metastasis, age, T stage, marital status, chemotherapy and surgery were independent prognostic factors affecting OS. Two nomograms predicting OS were established, with great discrimination (C-index between 0.67 and 0.81) and excellent calibration. Factors including age, race, sex, tumour grade, T stage, N stage, presence of liver, brain or bone metastasis, marital status, insurance status and anatomic location were related to the occurrence of lung metastasis in CRC patients.

**Conclusion:**

We developed two reliable clinical prediction models among CRC patients to predict the OS rates in patients with lung metastasis and lung metastasis only.

**Supplementary Information:**

The online version contains supplementary material available at 10.1186/s12876-022-02547-9.

## Introduction

Most people with colorectal caner (CRC) die from the disease, and it is the second leading cause of death in the United States [1]. There are approximately 1.8 million new cases of CRC and 900,000 related deaths each year [2]. Metastasis is a critical element in cancer-related deaths [3, 4]. Nearly half of patients with CRC will develop metastasis [5]. Approximately 21% of all CRC patients are diagnosed with stage IV disease. The areas that are most prone to metastasis are the liver and the lung [6]. The prognosis of CRC is related to the American Joint Committee on Cancer/International Union Against Cancer (AJCC/UICC) tumour-node-metastasis (TNM) staging system [7]. However, some literature highlights that, due to patient heterogeneity, the understanding of the prognosis of patients after lung metastasis treatment is beyond the scope of AJCC staging [8]. We need a more accurate prognostic system.

The lung is the second most common site of CRC metastasis, after the liver, accounting for approximately 10–15% of metastasis [9]. The median survival time for CRC patients with lung metastasis is 17.7 months (range, 5.9–31.2) [10]. We are also interested in the prediction of prognosis for those who have already developed lung metastasis. Prognosis evaluation experiments show that the 5-year overall survival (OS) rate of patients with lung metastasis is much better than that of patients with liver metastasis and brain metastasis (16.70, 15.99 and 5.51%, respectively) [11].

Some articles suggest that the site of the primary tumour also affects prognosis. That is, because of a more aggressive phenotype, right colon cancer has a worse prognosis [12–14]. In addition, there are some clinical differences between patients with left colon tumours and rectal cancers [15]. An article pointed out that R0 resection, preoperative carcinoembryonic antigen level, lymph node involvement rate and number of lesions are important factors affecting the prognosis of CRC patients with lung metastasis [16]. The nomogram is an intuitive display of a statistical prediction model that produces numerical probabilities of clinical events [17]. Nomograms are widely used to predict the survival of tumour patients.

Due to the lack of large-scale retrospective studies describing the clinical characteristics of CRC patients with lung metastasis, no prediction of cancer-specific survival (CSS) has been reported, and limited information is available to analyse the prognosis of CRC patients with lung-only metastasis. Therefore, we used information from the Surveillance, Epidemiology, and End Results (SEER) database to analyse the incidence, risk factors, and prognostic factors of CRC lung metastasis. This analysis was performed separately for CRC patients who developed lung metastasis and those who had only lung metastasis. Additionally, we reviewed the data of 70 CRC patients who were diagnosed with lung metastasis and were admitted to our hospital and analysed their clinical characteristics, treatment methods and efficacy.

## Materials and methods

### Data extraction

The SEER database is a comprehensive cancer statistics database that contains 17 population-based cancer registries that account for 28% of the US population and record information on individuals with malignant tumours in the United States. We first submitted the data consent form to the SEER administration and then collected the relevant data using SEER*Stat version 8.3.5. The information presented in this study is based on the most recent follow-up (May 30, 2020) available in the SEER database. Informed consent was not necessary in our investigation since the data gathered from the SEER database were anonymized prior to release.

### Data arrangement

**Fig. 1:**
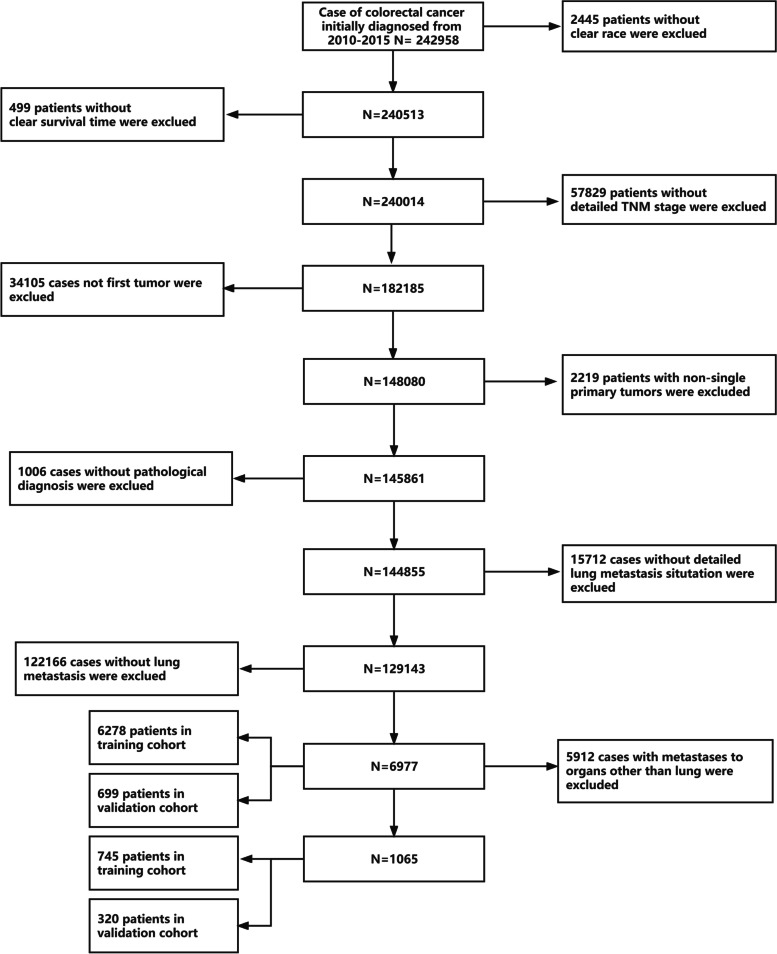
The flow diagram of the selection process for the study

New CRC cases were collected from the SEER database between January 2010 and December 2015. Based on these data, we chose patients using the procedure depicted in Fig. 1. The eligibility criteria were as follows: (1) no history of other tumours before CRC diagnosis; (2) a positive pathologic diagnosis; and (3) follow-up time of more than 1 year. The exclusion criteria were as follows: (1) unclear lung metastatic status; (2) previous tumour diagnosis; (3) no comprehensive tumour stage information; (4) unknown race; and (5) unknown survival time or survival time not coded. We gathered information from each patient record, including race, sex, age, original tumour site, degree of tumour differentiation, tumour size, AJCC stage, T stage, N stage, and the occurrence of distant metastasis affecting bone, brain, liver, or lung at the time of first CRC diagnosis. In addition, the patients’ survival statistics were obtained. All patients were classified into two groups: those with lung metastasis and those with lung-only metastasis but no metastasis in other organs. The two groups were then randomly divided into two cohorts (training cohorts and validation cohorts) and a 7:3 population was obtained. To further evaluate the performance of the predictive model, the CRC data from the SEER database for the period 2016–2019 was used as the external validation set (Supplemental Table 1).

### Patient data from our hospital

The study included 70 CRC patients with lung metastasis who underwent resection at Shanghai East Hospital between May 2016 and March 2019. The following criteria were used to determine inclusion: stage III-IV CRC; lung metastasis discovered after CRC resection in our institution; CT utilized to confirm lung metastasis; and more than 2 years of follow-up. Sex, age, site, T and N stage, survival months, organ metastasis, lymph node involvement, classification of lung metastasis, interval between operation and lung metastasis, treatment after lung metastasis, interval between lung metastasis and death or last follow-up, carcinoembryonic antigen (CEA) level (normal range, 0–5 ng/mL), and status were collected from each record. Due to the limited sample size, the cause of death for 70 patients at our hospital could not be determined. The 2016 ESMO handbook defines oligometastasis as “the presence of metastasis at up to two or three locations and five or more lesions.” This study followed the Declaration of Helsinki and the ethical principles of Shanghai East Hospital.

### Statistical analysis

For the description of baseline attributes, descriptive statistics are utilized. The *x*^2^-test was performed to analyse lung metastasis incidence between subgroups for categorical variables to identify risk factors for CRC patients with lung metastasis, and variables with statistical significance were chosen for multiple logistic regression. The Kaplan–Meier technique and log-rank test were used in the training cohort to determine factors that impacted OS; competitive risk analysis was utilized to identify significant variables that influenced CSS; and a multivariate analysis was performed using a Cox regression model. The concordance index (C-index) was used to examine the internal validity of the nomogram, which was calculated by comparing the nomogram-predicted probability with the observed probability. The nomogram was then verified further by comparing the nomogram-predicted likelihood of patients in the validation cohort to their actual survival. SPSS 24.0 (IBM Corp, Armonk, NY, USA) and the ggplot and rms packages in R 3.4.3 (https://www.r-project.org/) were used for statistical analyses. All statistical tests were two-tailed, with *p* < 0.05 deemed statistically significant.

## Results

### Demographic and clinical characteristics

As shown in Table 1, 129,170 CRC patients diagnosed between 2010 and 2015 were included. Out of 6977 patients who developed lung metastasis, 1065 had lung-only metastasis. The median age of the 6977 patients was 65 years, with pathological grade II accounting for 65.5%, and the lesions were mostly located in the right-sided colon (40.9%). The median age of the 1065 patients with only lung metastasis was 66 years. The majority of the population (58.8%) had stage II CRC, and the lesions were mostly located in the rectum (40.5%).

**Table 1:** Clinical and demographic characteristics of the patient cohort

Characteristic	All patients	Patients with lung metastasis	Patients without lung metastasis	Patients with lung-only metastasis
	*N* = 129,143	*N* = 6977	*N* = 122,166	*N* = 1065
**Age**				
< 65	63,024 (48.8)	3724 (53.4)	59,300 (48.5)	479 (45.0)
≥ 65	66,119 (51.2)	3253 (46.6)	62,866 (51.5)	586 (55.0)
**Race**				
White	100,788 (78.0)	5124 (73.4)	95,664 (78.3)	798 (74.9)
Black	15,650 (12.2)	1101 (15.8)	14,549 (11.9)	159 (14.9)
Others	12,705 (9.8)	752 (10.8)	11,953 (9.8)	108 (10.2)
**Gender**				
Female	61,569 (47.7)	3146 (45.1)	58,423 (47.8)	518 (48.6)
Male	67,574 (52.3)	3831 (54.9)	63,743 (52.2)	547 (51.4)
**Grade**				
Grade I	10,168 (7.9)	313 (4.5)	9855 (8.1)	51 (4.8)
Grade II	84,526 (65.5)	3715 (53.2)	80,811 (66.1)	626 (58.8)
Grade III	19,129 (14.8)	1034 (14.8)	18,095 (14.8)	140 (13.1)
Grade IV	3679 (2.8)	171 (2.5)	3508 (2.9)	23 (2.2)
Unknown	11,641 (9.0)	1744 (25.0)	9897 (8.1)	225 (21.1)
**AJCC TNM stage**				
I	30,460 (23.6)	0 (0.0)	30,460 (24.9)	0 (0.0)
II	33,081 (25.6)	0 (0.0)	33,081 (27.1)	0 (0.0)
III	37,915 (29.4)	0 (0.0)	37,915 (31.0)	0 (0.0)
IV	27,687 (21.4)	6977 (100.0)	20,710 (17.0)	1065 (100.0)
**AJCC T stage**				
T0	56 (0.0)	9 (0.1)	47 (0.0)	3 (0.3)
T1	22,947 (17.8)	904 (13.0)	22,043 (18.0)	125 (11.7)
T2	16,173 (12.5)	130 (1.9)	16,043 (13.2)	37 (3.5)
T3	62,061 (48.1)	2028 (29.0)	60,033 (49.1)	492 (46.2)
T4	21,021 (16.3)	1606 (23.0)	19,415 (15.9)	242 (22.7)
TX	6885 (5.3)	2300 (33.0)	4585 (3.8)	166 (15.6)
**AJCC N stage**				
N0	71,956 (55.7)	2317 (33.2)	69,639 (57.0)	471 (44.2)
N1	35,029 (27.2)	2369 (34.0)	32,660 (26.7)	394 (37.0)
N2	19,388 (15.0)	1293 (18.5)	18,095 (14.8)	200 (18.8)
N3	2770 (2.1)	998 (14.3)	1772 (1.5)	0 (0.0)
**AJCC M stage**				
M0	101,456 (78.6)	0 (0)	101,456 (83.0)	0 (0.0)
M1	27,687 (21.4)	6977 (100.0)	20,710 (17.0)	1065 (100.0)
**Marital status**				
Married	67,504 (52.3)	3299 (47.3)	64,205 (52.6)	489 (45.9)
Single	53,026 (41.0)	3203 (45.9)	49,823 (40.8)	521 (48.9)
Unknown	8613 (6.7)	475 (6.8)	8138 (6.6)	55 (5.2)
**Insurance status**				
Insured	84,167 (65.2)	4002 (57.4)	80,165 (65.6)	1006 (94.5)
Uninsured	4693 (3.6)	401 (5.7)	4292 (3.5)	45 (4.2)
Unknown	40,283 (31.2)	2574 (36.9)	37,709 (30.9)	14 (1.3)
**Chemotherapy**				
Yes	57,583 (44.6)	4700 (67.4)	52,883 (43.3)	730 (68.5)
No/Unknown	71,560 (55.4)	2277 (32.6)	69,283 (56.7)	335 (31.5)
**Site**				
Right-sided colon	52,861 (40.9)	2168 (31.1)	50,693 (41.5)	304 (28.5)
Left-sided colon	36,085 (27.9)	1854 (26.6)	34,231 (28.0)	219 (20.6)
Rectum	29,231 (22.6)	2165 (31.0)	27,066 (22.2)	431 (40.5)
Rectosigmoid	10,966 (8.6)	790 (11.3)	10,176 (8.3)	111 (10.4)
**Surgery**				
No	16,789 (13.0)	4305 (61.7)	12,461 (10.2)	475 (44.6)
Yes	111,450 (86.3)	2595 (37.2)	108,850 (89.1)	586 (55.0)
Unknown	904 (0.7)	77 (1.1)	855 (0.7)	4 (0.4)
**Radiotherapy**				
Yes	21,396 (16.6)	1164 (16.7)	20,232 (16.6)	267 (25.1)
No/Unknown	107,747 (83.4)	5813 (83.3)	101,934 (83.4)	798 (74.9)
**Osseous metastasis**				–
No	127,287 (98.6)	6107 (87.6)	121,180 (99.2)	
Yes	1511 (1.1)	672 (9.6)	839 (0.7)	
Unknown	345 (0.3)	198 (2.8)	147 (0.1)	
**Brain metastasis**				–
No	128,368 (99.4)	6564 (94.0)	121,804 (99.8)	
Yes	367 (0.3)	185 (2.7)	182 (0.1)	
Unknown	408 (0.3)	228 (3.3)	180 (0.1)	
**Liver metastasis**				–
No	108,363 (83.9)	1892 (27.2)	106,471 (87.2)	
Yes	20,641 (16.0)	5019 (71.9)	15,622 (12.7)	
Unknown	139 (0.1)	66 (0.9)	73 (0.1)	

### Risk of factors for developing LM among CRC patients

As illustrated in Supplemental Table 2, age (*p* < 0.001); race (*p* < 0.001); sex (*p* < 0.001); grade (*p* < 0.001); primary tumour site (*p* < 0.001); T stage (*p* < 0.001); N stage (*p* < 0.001); the presence of liver (*p* < 0.001), brain (*p* < 0.001), or bone (*p* < 0.001) metastasis at initial diagnosis; marital status (*p* = 0.004) and insurance status (*p* < 0.001) were independent prognostic factors that affected the risk of lung metastasis.

### Survival analysis

**Fig. 2:**
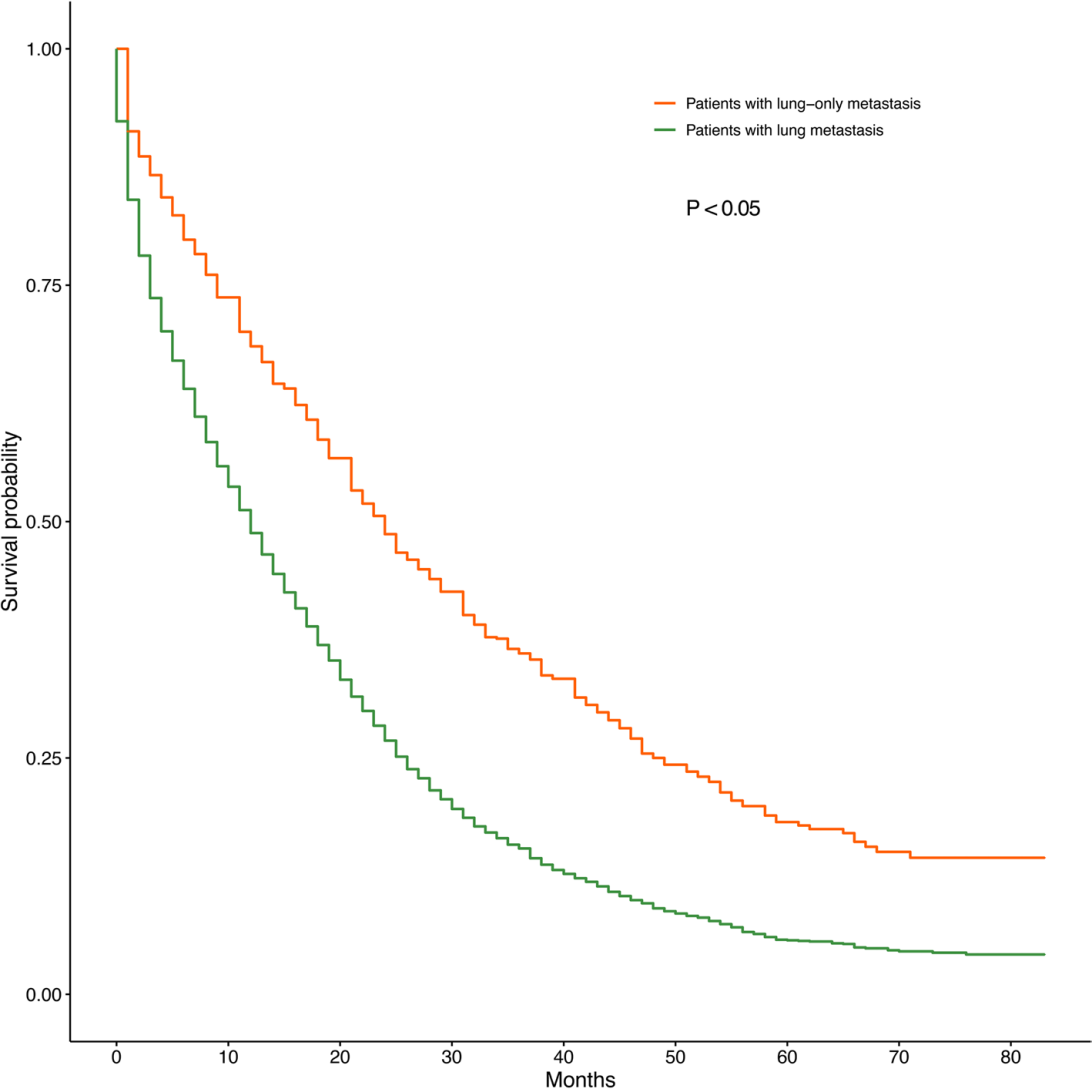
Overall Kaplan-Meier survival curves for CRC patients with lung metastasis and lung-only metastasis

Table 2 shows that 6278 of the 6977 patients were on the training cohort list. The median survival time in the training cohort was 12 months (range, 11.4–12.6 months), with a 1-year OS of 49.2% ± 0.6% and a 3-year OS of 15.9% ± 0.5%. Another 745 of the 1065 CRC patients with isolated lung metastasis were enrolled as the training set. The median survival time in the training set was 24 months (range, 11.8–26.1 months), with 1-year and 3-year OS rates of 68.5 ± 1.4 and 36.1% ± 1.6%, respectively. Kaplan–Meier survival analysis was applied to compare the difference in OS between the two groups of CRC patients with lung metastasis and lung-only metastasis (Fig. 2).

**Table 2:** 1- and 3-year medium survival rates and univariate analysis of patients with lung metastasis in colorectal cancer

Variable	Patients with lung metastasis (*N* = 6278)				Patients with lung-only metastasis (*N* = 1065)			
	1-yearOS (%)	3-year OS (%)	Median survival time (months)	*p* value	1-yearOS (%)	3-year OS (%)	Median survival time (months)	*p* value
**Total case**	49.2 ± 0.6	15.9 ± 0.5	12.0 (11.4–12.6)		68.5 ± 1.4	36.1 ± 1.6	24.0 (21.8–26.1)	
**Age**				< 0.001				< 0.001
< 65	57.8 ± 0.9	18.8 ± 0.8	17.0 (16.2–17.8)		81.1 ± 1.8	49.6 ± 2.6	35.0 (29.7–40.2)	
≥ 65	38.5 ± 0.9	11.5 ± 0.7	8.0 (7.3–8.7)		58.2 ± 0.2	25.0 ± 2.0	17.0 (14.8–19.2)	
**Grade**				< 0.001				< 0.001
Grade I	54.8 ± 3.1	19.3 ± 2.8	15.0 (11.9–18.1)		61.1 ± 8.1	28.5 ± 8.6	25.0 (21.2–28.8)	
Grade II	56.3 ± 0.9	19.5 ± 0.8	15.0 (14.1–15.9)		75.1 ± 2.1	41.4 ± 2.7	31.0 (27.4–34.6)	
Grade III	36.9 ± 1.6	11.2 ± 1.2	7.0 (5.9–8.1)		58.3 ± 5.0	35.2 ± 5.2	18.0 (10.9–25.0)	
Grade IV	36.5 ± 4.0	13.8 ± 2.3	7.0 (4.2–9.8)		74.0 ± 1.2	–	24.0 (2.6–45.4)	
Unknown	41.7 ± 1.2	10.2 ± 0.9	9.0 (8.0–9.9)		51.3 ± 3.8	–	14.0 (10.0–18.0)	
**AJCC T stage**				< 0.001				< 0.001
T0	43.9 ± 1.8	11.1 ± 1.3	1.0 (0–2.5)		–	–	6.0 (3.1–10.2)	
T1	66.4 ± 4.3	29.5 ± 4.7	10.0 (8.6–11.4)		57.5 ± 5.3	–	21.0 (11.2–30.8)	
T2	62.5 ± 1.1	26.0 ± 1.2	21.0 (14.9–27.1)		67.9 ± 8.8	57.8 ± 1.0	41.0 (29.7–52.3)	
T3	26.6 ± 1.2	12.0 ± 1.0	19.0 (17.9–20.1)		78.2 ± 2.2	46.6 ± 3.0	33.0 (27.8–38.2)	
T4	15.4 ± 1.1	5.3 ± 0.9	12.0 (10.9–13.0)		58.1 ± 3.9	28.2 ± 3.0	19.0 (12.9–25.0)	
T_X_	–	–	8.0 (7.2–8.8)		46.9 ± 4.7	11.8 ± 3.8	11.0 (6.0–16.0)	
**AJCC N stage**				< 0.001				< 0.001
N0	47.7 ± 1.1	15.5 ± 0.9	12.0 (11.0–13.0)		61.4 ± 2.3	28.4 ± 2.3	19.0 (16.0–22.0)	
N1	53.0 ± 1.1	18.9 ± 1.0	14.0 (12.9–15.1)		74.7 ± 2.2	43.0 ± 2.8	29.0 (24.2–34.0)	
N2	54.6 ± 1.5	17.3 ± 1.0	14.0 (12.6–15.4)		73.3 ± 3.1	39.6 ± 3.0	26.0 (21.9–26.1)	
N3	36.7 ± 1.6	7.9 ± 1.0	7.0 (5.8–8.2)		–	–	–	
**Marital status**				< 0.001				< 0.001
Married	54.2 ± 0.9	18.7 ± 0.8	14.0 (13.2–14.8)		60.4 ± 2.2	29.6 ± 2.2	19.0 (16.4–21.6)	
Single	44.1 ± 0.9	12.6 ± 0.7	10.0 (9.3–10.7)		77.7 ± 1.9	42.7 ± 2.5	31.0 (27.1–34.9)	
Other	48.5 ± 2.5	17.9 ± 2.1	12.0 (9.5–14.5)		63.6 ± 6.5	29.7 ± 6.5	22.0 (21.9–26.1)	
**Chemotherapy**				< 0.001				< 0.001
Yes	63.7 ± 0.7	21.0 ± 0.7	18.0 (17.3–18.7)		83.0 ± 1.4	47.6 ± 2.2	33.0 (29.8–36.2)	
No/Unknown	18.6 ± 0.9	5.0 ± 0.6	2.0 (1.7–2.3)		36.7 ± 2.7	11.2 ± 1.9	8.0 (6.3–9.7)	
**Radiotherapy**				< 0.001				< 0.001
Yes	58.5 ± 1.5	21.9 ± 1.4	16.0 (14.8–17.2)		78.6 ± 2.5	43.6 ± 3.3	31.0 (25.1–36.9)	
No/Unknown	46.9 ± 0.7	14.1 ± 0.6	11.0 (10.4–11.6)		65.2 ± 1.7	33.1 ± 1.9	21.0 (18.8–23.2)	
**Surgery**				< 0.001				< 0.001
Yes	61.7 ± 1.0	25.7 ± 1.0	19.0 (17.8–20.2)		79.4 ± 1.7	50.1 ± 2.3	37.0 (33.0–40.9)	
No/Unknown	41.7 ± 0.8	9.7 ± 0.6	9.0 (8.4–9.6)		55.3 ± 2.3	17.9 ± 2.1	14.0 (11.9–16.1)	
**Site**				< 0.001				0.002
Right-sided colon	39.1 ± 1.1	9.7 ± 0.8	8.0 (7.2–8.8)		58.8 ± 2.8	29.3 ± 2.8	19.0 (16.4–21.6)	
Left-sided colon	51.2 ± 1.2	17.8 ± 1.1	13.0 (11.9–14.1)		72.1 ± 3.1	40.2 ± 3.7	27.0 (22.4–31.6)	
Rectum	56.4 ± 1.1	19.4 ± 1.0	15.0 (13.9–16.1)		71.1 ± 4.3	41.2 ± 5.6	31.0 (21.4–40.6)	
Rectosigmoid	52.3 ± 1.9	18.8 ± 1.7	14.0 (12.1–15.9)		72.9 ± 2.1	37.0 ± 2.6	25.0 (21.4–28.6)	
**Insurance status**				< 0.001				0.042
Insured	52.6 ± 0.8	18.1 ± 0.7	14.0 (13.2–14.8)		72.8 ± 6.7	24.7 ± 7.2	21.0 (16.3–25.7)	
Uninsured	48.0 ± 2.7	14.1 ± 2.1	12.0 (9.8–14.2)		68.4 ± 1.5	36.7 ± 1.7	24.0 (21.6–26.4)	
Unknown	44.1 ± 1.0	12.7 ± 12.8	10.0 (9.2–10.8)		64.3 ± 12	–	22.0 (21.9–26.1)	
**Race**				0.027				0.218
White	49.4 ± 0.7	16.3 ± 0.6	12.0 (11.3–12.7)		73.3 ± 3.5	33.7 ± 3.5	23.0 (20.6–25.2)	
Black	46.1 ± 1.6	12.4 ± 1.2	11.0 (9.8–12.2)		67.2 ± 1.7	35.5 ± 1.9	25.0 (20.0–30.0)	
Others	48.3 ± 1.9	6.0 ± 1.4	12.0 (10.2–13.7)		71.3 ± 0.4	36.5 ± 5.7	29.0 (21.8–36.2)	
**Gender**				0.946				0.271
Female	48.2 ± 0.9	16.1 ± 0.8	12.0 (11.112.9)		65.3 ± 2.5	34.6 ± 2.8	23.0 (19.8–26.2)	
Male	49.4 ± 0.9	14.5 ± 0.7	12.0 (11.3–12.7)		68.3 ± 2.4	36.6 ± 2.7	24.0 (20.5–27.5)	
**Osseous metastasis**				< 0.001	–	–	–	
No	51.4 ± 0.7	17.1 ± 0.6	13.0 (12.4–13.6)					
Yes	31.0 ± 0.9	6.4 ± 1.2	6.0 (4.9–7.1)					
Unknown	41.8 ± 3.8	10.6 ± 2.5	7.0 (5.3–8.7)					
**Brain metastasis**				< 0.001	–	–	–	
No	17.1 ± 0.6	16.4 ± 0.6	13.0 (12.4–13.6)					
Yes	25.6 ± 3.4	11.1 ± 1.3	4.0 (2.4–5.6)					
Unknown	60..9 ± 6.4	19.0 ± 5.4	17.0 (12.7–21.3)					
**Liver metastasis**				< 0.001	–	–	–	
No	49.7 ± 0.7	15.9 ± 0.5	20.0 (18.5–21.5)					
Yes	24.9 ± 3.4	–	10.0 (9.4–10.6)					
Unknown	41.2 ± 3.5	11.3 ± 2.4	17.0 (12.7–21.3)

**Fig. 3:**
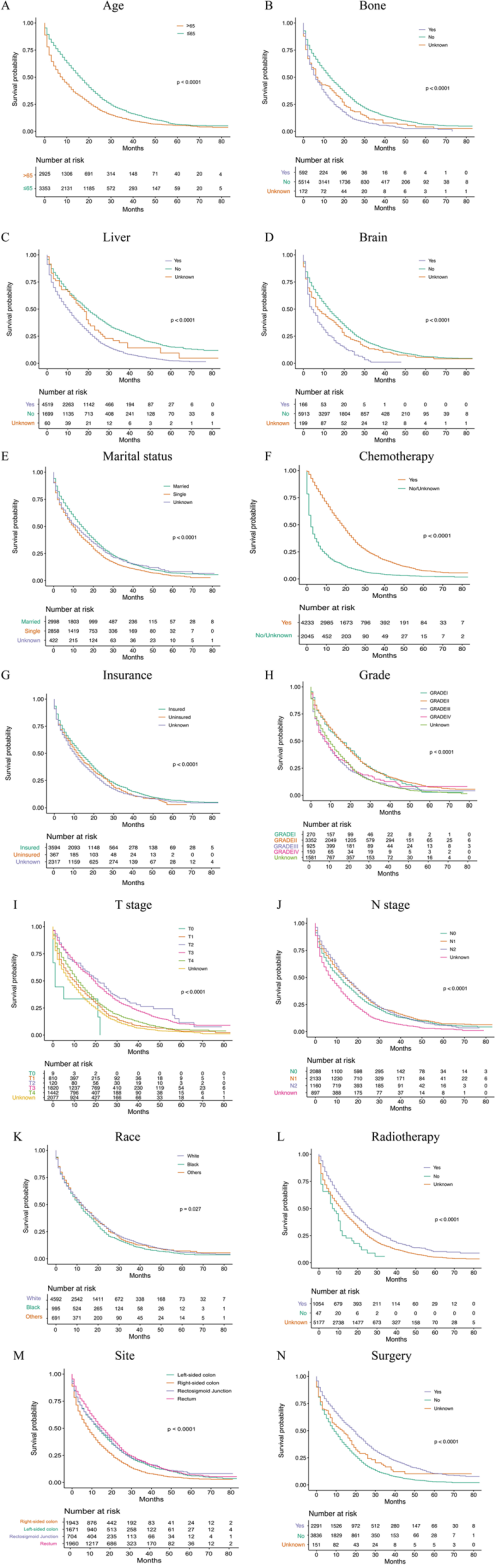
1 Overall Kaplan-Meier survival curves for CRC patients with lung metastasis in training cohort according to **A** Age, **B** Bone metastasis, **C** Liver metastasis, **D** Brain metastasis, **E** Marital status, **F** Chemotherapy, **G** Insurance status, **H** Grade. 2 Overall Kaplan-Meier survival curves for CRC patients with lung metastasis in training cohort according to **I** T stage, **J** N stage, **K** Race, **L** Radiotherapy, **M** Site, **N** Surgery

In the colorectal cancer lung metastasis group, the Kaplan–Meier analysis results are delineated in Fig. 3, which demonstrates that age (*p* < 0.001); race (*p* = 0.027); tumour grade (*p* < 0.001); T stage (*p* < 0.001); N stage (*p* < 0.001); M stage (*p* < 0.001); bone metastasis (*p* < 0.001); brain metastasis (*p* < 0.001); liver metastasis (*p* < 0.001); marital status (*p* < 0.001); insurance status (*p* < 0.001); chemotherapy (*p* < 0.001); grade (*p* < 0.001); radiation therapy (*p* < 0.001); surgery (*p* < 0.001); and primary tumour site (*p* < 0.001) were significant prognostic factors. Moreover, competing risk models were constructed, and the CSS curves of the patients were parsed (Supplemental Fig. 1).

**Fig. 4:**
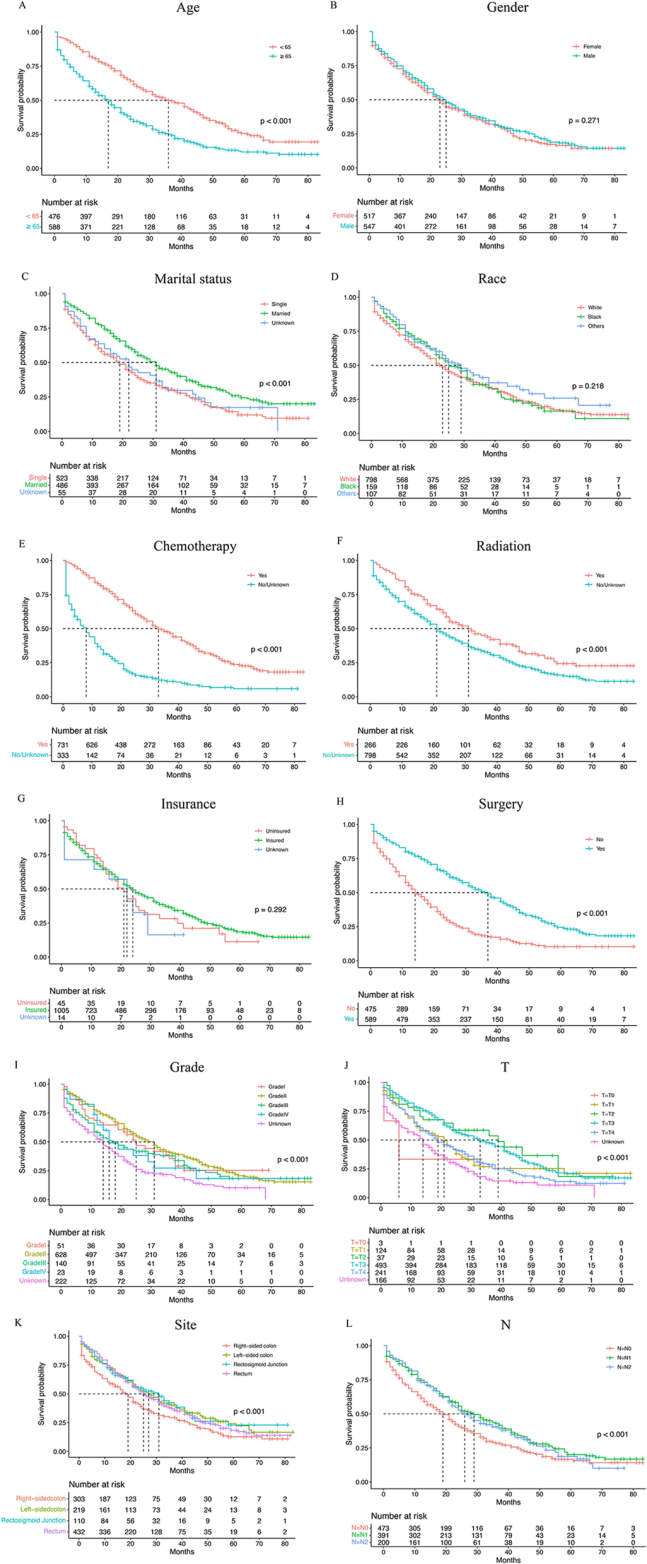
1 Overall Kaplan-Meier survival curves for CRC patients with lung-only metastasis in training cohort according to **A** Age, **B** Gender, **C** Marital status, **D** Race, **E** Chemotherapy, **F** Radiotherapy, **G** Insurance status, **H** Surgery. 2 Overall Kaplan-Meier survival curves for CRC patients with lung-only metastasis in training cohort according to **I** Grade, **J** T stage, **K** Site, **L** N stage

As shown in Table 3, several variables were independently associated with OS, as follows: age (*p* < 0.001); pathological grade (*p* < 0.001); T stage (*p* < 0.001); N stage (*p* < 0.001); bone metastasis (*p* < 0.001); brain metastasis (*p* < 0.001); liver metastasis (*p* < 0.001); primary tumour site (*p* < 0.001) and surgery (*p* < 0.001). Additionally, the survival outcomes among CRC patients with isolated lung metastasis were as follows. The results of the Kaplan–Meier analysis are presented in Fig. 4. In Table 4, we screened the following independent prognostic factors affecting the prognosis of CRC patients with only lung metastasis: age (*p* < 0.001); T stage (*p* < 0.001); marital status (*p* < 0.001); chemotherapy (*p* < 0.001) and surgery (*p* < 0.001).

**Table 3:** Multiple COX regression results of OS and Non-cancer-specific survival (NCCSS) in CRC patients with lung metastasis

Independent prognostic factors	OR	95%CI	*P*
**OS**			
**Age**			< 0.001
< 65	1		
≥ 65	1.242	1.156–1.335	
**AJCC T stage**			< 0.001
T0	1		
T1	1.119	0.358–3.502	0.847
T2	0.941	0.293–3.019	0.918
T3	0.922	0.295–2.884	0.889
T4	1.186	0.379–3.710	0.770
Tx	1.067	0.342–3.330	0.911
**AJCC N stage**			0.012
N0	1		
N1	1.038	0.950–1.134	0.410
N2	1.175	1.046–1.319	0.007
N3	1.135	1.012–1.274	0.031
**Osseous metastasis**			< 0.001
No	1		
Yes	1.601	1.429–1.793	< 0.001
Unknown	1.249	0.892–1.750	0.195
**Brain metastasis**			< 0.001
No	1		
Yes	1.648	1.321–2.058	< 0.001
Unknown	0.926	0.678–1.264	0.628
**Liver metastasis**			< 0.001
No	1		
Yes	1.670	1.532–1.821	< 0.001
Unknown	1.136	0.759–1.699	< 0.001
**Site**			< 0.001
Right-sided colon	1		
Left-sided colon	0.765	0.696–0.840	< 0.001
Rectum	0.679	0.618–0.745	< 0.001
Rectosigmoid Junction	0.703	0.621–0.796	< 0.001
**Surgery**			< 0.001
Yes	1		
No/Unknown	1.519	1.414–1.632	
**Grade**			< 0.001
Grade I	1		
Grade II	1.108	0.922–1.332	0.272
Grade III	1.541	1.262–1.881	< 0.001
Grade IV	1.539	1.156–2.049	0.003
Unknown	1.171	0.968–1.418	0.104
**Non-cancer-specific survival**			
**Age**			< 0.001
< 65	1		
≥ 65	1.926	1.483–2.502	
**AJCC T stage**			< 0.001
T0	1		
T1	0.136	0.358–3.502	0.006
T2	0.182	0.293–3.019	0.027
T3	0.110	0.295–2.884	0.002
T4	0.115	0.379–3.710	0.003
Tx	0.187	0.342–3.330	0.019
**Chemotherapy**			< 0.001
Yes	1		
No/Unknown	3.185	2.520–4.024	
**Marital status**			< 0.001
Married	1		
Single	1.757	1.380–2.237	< 0.001
Unknown	2.392	1.651–3.467	< 0.001

**Table 4:** Univariate and multivariate Cox regression analysis results of OS in patients with lung-only metastasis

Characteristic	No. of CRC patients with lung-only metastasis (*N* = 1065) (%)	Univariable			Multivariable		
		OR	95%CI	*p* value	OR	95%CI	*p* value
**Age**				< 0.001			< 0.001
< 65	479(45.0)	1			1		
≥ 65	586(55.0)	1.994	1.709–2.327		1.555	1.320–1.833	
**AJCC T stage**				< 0.001			< 0.001
T0	3(0.3)	1			1		
T1	125(11.7)	0.726	0.178–2.950	0.654	0.954	0.233–3.906	0.947
T2	37(3.5)	0.435	0.102–1.862	0.262	1.005	0.231–4.370	0.994
T3	492(46.2)	0.481	0.120–1.932	0.302	1.102	0.271–4.489	0.892
T4	242(22.7)	0.750	0.186–3.023	0.685	1.805	0.443–7.356	0.410
T_X_	166(15.6)	1.064	0.263–4.034	0.930	1.220	0.300–4.963	0.781
**Marital status**				< 0.001			< 0.001
Single	521(48.9)	1			1		
Married	489(45.9)	0.639	0.548–0.747	< 0.001	0.723	0.617–0.849	< 0.001
Other	55(5.2)	0.904	0.657–1.244	0.536	0.768	0.557–1.059	0.107
**Chemotherapy**				< 0.001			< 0.001
Yes	730(68.5)	1			1		
No/Unknown	335(31.5)	3.382	2.900–3.943		2.904	2.463–3.423	
**Surgery**				< 0.001			< 0.001
No	479(45.0)	1			1		
Yes	586(55.0)	0.445	0.382–0.519	< 0.001	0.477	0.393–0.578	< 0.001
**Grade**				< 0.001			0.016
Grade I	51(4.8)	1			1		
Grade II	626(58.8)	0.872	0.611–1.245	0.452	0.903	0.631–1.292	0.576
Grade III	140(13.1)	1.166	0.785–1.733	0.446	1.267	0.850–1.889	0.245
Grade IV	23(2.2)	1.198	0.659–2.177	0.553	1.214	0.665–2.216	0.529
Unknown	225(21.1)	1.709	1.178–2.480	0.005	1.153	0.785–1.693	0.467
**Radiotherapy**				< 0.001			–
Yes	267(25.1)	1					
No/Unknown	798(74.9)	1.467	1.228–1.752				
**AJCC N stage**				< 0.001			–
N0	471(44.2)	1			1		
N1	394(37.0)	0.705	0.597–0.833	< 0.001			
N2	200(18.8)	0.751	0.612–0.922	0.006			
**Site**				< 0.001			–
Right-sided colon	304(28.5)	1			1		
Left-sided colon	219(20.6)	0.698	0.564–0.864	0.001			
Rectosigmoid	111(10.4)	0.662	0.500–0.875	0.004			
Rectum	431(40.5)	0.735	0.616–0.877	0.001			
**Race**				0.231			–
White	798(74.9)	–			1		
Black	159(14.9)			0.682			
Others	108(10.0)			0.113			
**Gender**				0.280			
Female	518(48.6)	–			1		–
Male	547(51.4)						
**Insurance status**				0.306			–
Uninsured	45(4.2)	–			1		
Insured	1006(94.5)			0.182			
Unknown	14(1.3)			0.201			

### Construction and validation of the nomogram

**Fig. 5:**
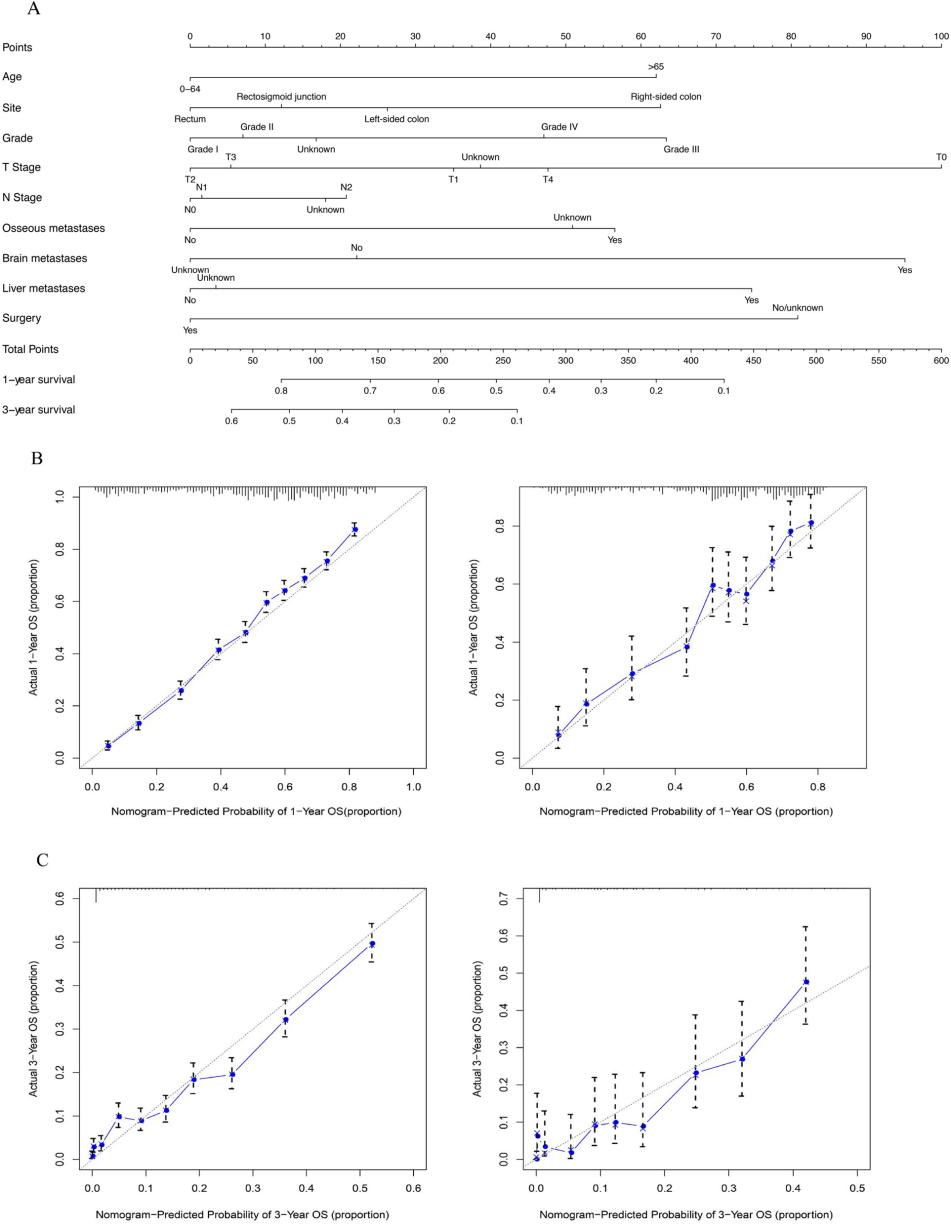
A nomogram for prediction of 1- and 3-year OS rates of CRC patients with lung metastasis (**A**); Calibration curve of the nomogram predicting 1- and 3-year OS rates of CRC patients with lung metastasis in training cohort (**B**); Calibration curve of the nomogram predicting 1- and 3-year OS rates of CRC patients with lung metastasis in the validation cohort (**C**)

**Fig. 6:**
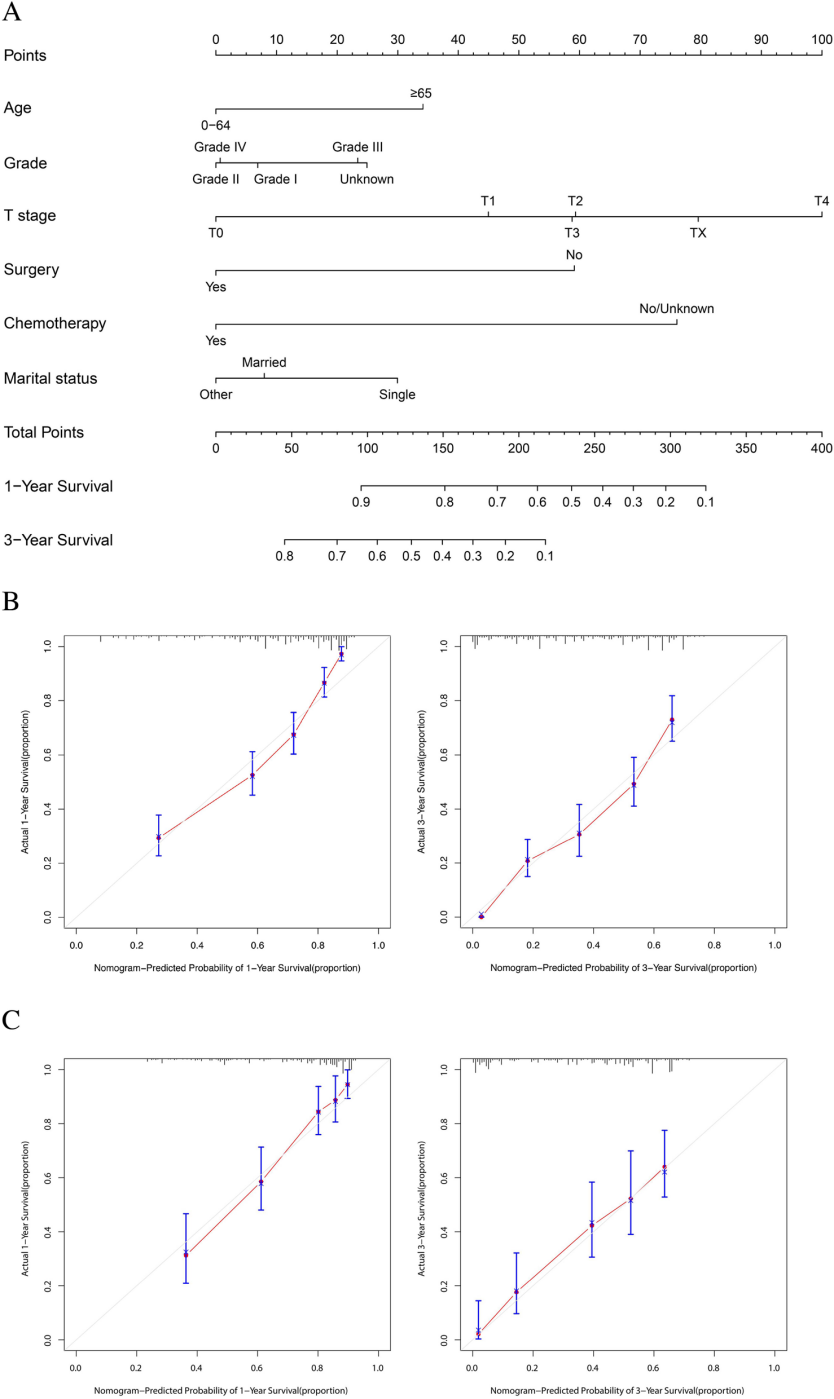
A nomogram for prediction of 1- and 3-year OS rates of CRC patients with lung-only metastasis (**A**); Calibration curve of the nomogram predicting 1- and 3-year OS rates of CRC patients with lung-only metastasis in training cohort (**B**); Calibration curve of the nomogram predicting 1- and 3-year OS rates of CRC patients with lung-only metastasis in the validation cohort (**C**)

As shown in Fig. 5, the final nomogram was developed for all colorectal cancer lung metastasis cases, depicting the 1- and 3-year OS by weighting the score of each variable. The accuracy of the nomogram was validated internally and externally using the identification and calibration method, and the calculated C-index was 0.67 for each. Further external validation was performed by applying data from SEER data for the years 2016–2019, involving a total of 2365 cases, and the C-index was 0.71(Supplemental Fig. 2). The nomogram was created incorporating six variables for CRC patients with lung metastasis only, as shown in Fig. 6. The nomogram was validated internally and externally, and the C-index of the resulting ROC curve was 0.81 for each. Further external validation was performed involving a total of 565 cases with the C-index of 0.76 for both, as shown in Supplemental Fig. 2.

### Clinical characteristics of patients with lung metastasis in our centre

**Fig. 7:**
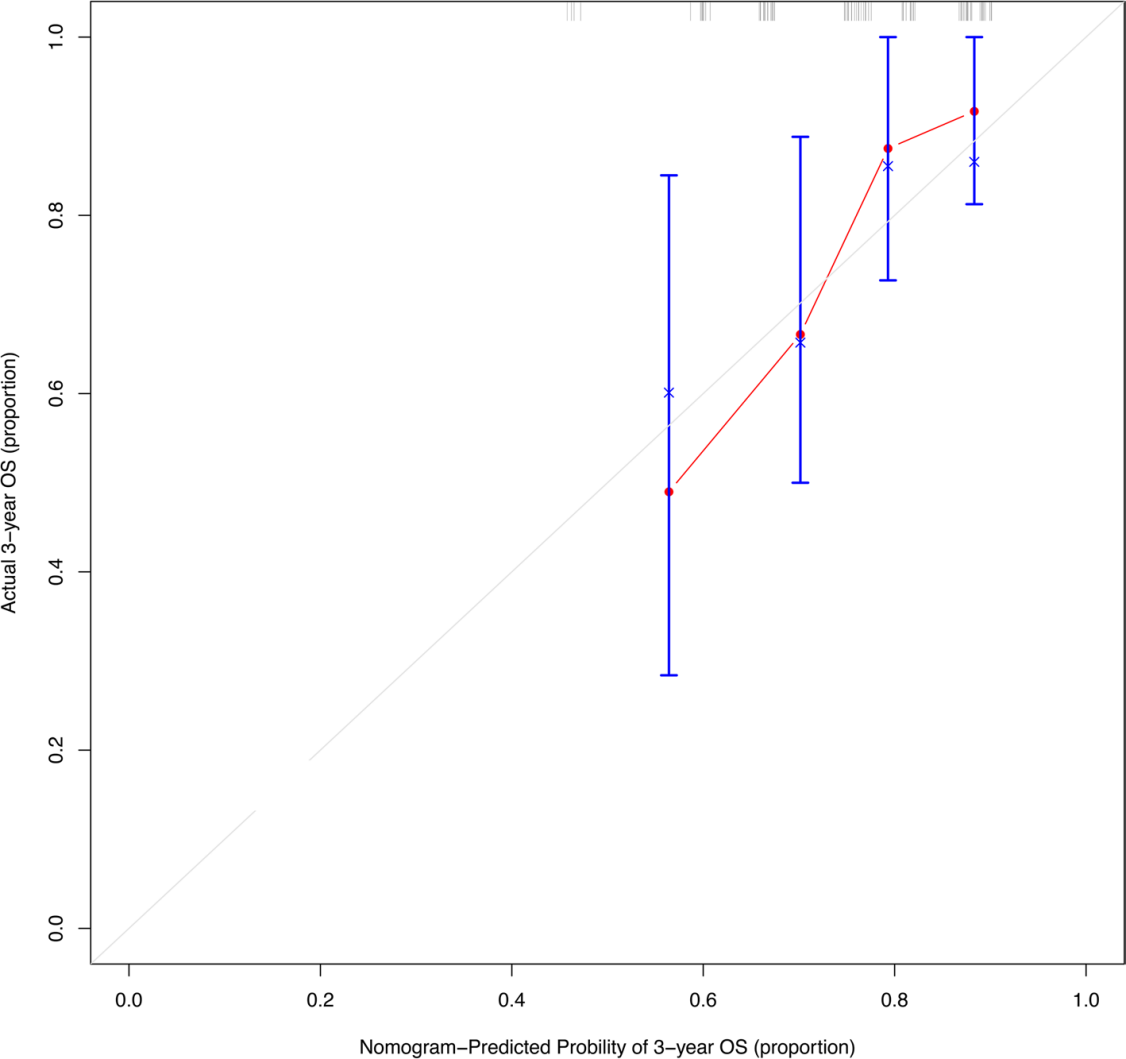
Calibration curve of the nomogram predicting 3-year OS rates of patients with lung metastasis in colorectal cancer in our hospital

Supplemental Table 3 presents detailed clinical data from 70 CRC patients with lung metastasis who underwent radical surgery at our centre. The mean CEA concentration (ng/mL) was 34 (range: 1–220). The median time between the start of therapy and the onset of lung metastasis was 14 months (range 1 to 106 months). From diagnosis to lung metastasis, the median period was 17 months. Within 2 years of surgery, metastasis developed in 47 instances. Twenty-three of the 70 individuals had only one pulmonary metastasis, whereas the remainder had numerous pulmonary metastasis. Thirteen of the 70 patients died during the follow-up period. The median time between pulmonary metastasis and death or final follow-up was comparable in patients with metastasis to multiple locations and individuals with only pulmonary metastasis (28 months vs. 29 months). In terms of treatment regimens, surgical resection was chosen for 8 patients, radiotherapy was chosen for 47 patients, chemotherapy was chosen for 54 patients. As illustrated in Fig. 7, data from this group of patients were entered into the existing nomogram that predicts OS as an external verification set to validate the 3-year OS rate and the C-index was 0.69.

## Discussion

This study assessed the independent prognostic factors affecting survival in 6278 CRC patients with lung metastasis and 1065 patients with only lung metastasis from the SEER database records of CRC diagnosed from 2010 to 2015. Two nomograms were separately established, which performed well in predicting survival. The data of 70 hospitalized patients diagnosed between May 2016 and March 2019 were collected and analysed using the nomogram. Data from patients with colorectal cancer lung metastasis and lung-only metastasis diagnosed between 2016 and 2019 selected from the SEER database were used as external validation cohorts.

Previous studies have predicted the 1- and 3-year total survival in patients with lung metastasis from CRC, but the variables included in these equations are different due to a lack of univariate analysis [18]. Furthermore, there is an important problem that has not received attention, which is that patients with lung metastasis from colorectal cancer are prone to complicated metastasis from other sites, such as the liver, brain, and bone. If only colorectal cancer pulmonary metastasis is analysed without excluding the combined metastasis of other organs, it will affect the judgement of survival of people with colorectal cancer pulmonary metastasis. In our study, of the 6278 patients with lung metastasis from CRC, liver metastasis accounted for 71.9%, bone metastasis accounted for 9.7% and brain metastasis accounted for 2.7%. To further explore the factors independently affecting the prognosis of patients with pulmonary metastasis, we examined 1065 patients with only pulmonary metastasis to fill this gap. In addition, most articles have focused on analysing OS and CSS, but 6.2% of patients who died did not die of cancer. We focused on this population and performed survival analyses.

In clinical practice, any organ metastasis (such as brain or bone metastasis) corresponds with a relatively poor prognosis [19]. According to previous reports, liver metastasis is a useful independent parameter for predicting the survival of patients with stage IV CRC [20]. According to the results of our study, it was clearly observed that the occurrence of liver, brain, and bone metastasis were all independent prognostic factors. In the nomogram, any organ metastasis led to a higher total score and lower long-term OS.

Different primary sites of tumours determine the different biological behaviours of tumours. In our study, the right colon was the most common site of colon cancer (40.9%), while rectal cancer accounted for 22.6% of cases. In terms of lung metastasis, the incidence of lung metastasis and the incidence of lung-only metastasis in the right colon compared to rectal cancer was 31.1% vs. 31 and 28.5% vs. 40.5%, respectively, which indicates a higher risk of lung metastasis in rectal cancer. This result is similar to findings from other studies [21]. Previous studies have shown shorter disease-free survival in patients with rectal cancer due to direct spread to the systemic circulation through the haemorrhoidal vein [22, 23]. Regarding the prognosis of patients, the results showed that the median survival times for patients with lung metastasis and lung-only metastasis in the rectal, left-sided colon, and right-sided colon were 15 months vs. 13 months vs. 8.0 months and 31 months vs. 27 months vs. 19 months, respectively. Patients with metastatic rectal cancer showed better survival outcomes than patients with metastatic colon cancer, and the conclusion are also obtained in a previous study [24]. Primary CRC tumours located in the rectum often lead to lung metastasis, but their OS is better than that of CRC tumours located in other parts of the colon, and in turn, the prognosis is better in metastatic left-sided colon than in right-sided colon. There is increasing evidence that right colon cancer has a more aggressive phenotype and leads to a worse prognosis than other cancers [12, 13]. The clinical difference between patients with right- and left-sided colon cancers has been observed, and the patients with right-sided colon cancer were significantly older, mostly females, and had a higher incidence of complications [14]. This may explain the poor survival of patients with right-sided colon cancer.

In terms of treatment, surgery is also a key factor affecting the prognosis of cancer. The choice of primary tumour surgery remains controversial. In fact, more than two-thirds of elderly patients with stage IV CRC are known to have undergone primary tumour surgery [25]. The reason for this is that primary tumours may promote the development of metastasis and have serious complications that can significantly reduce the survival time of patients [26–28]. Moreover, resection of the primary tumour may contribute to the recovery of autoimmunity [27]. Some articles have confirmed that primary tumour surgery is associated with an increase in OS [29]. However, considering that complications from surgery delay the overall treatment plan [30] and that only a small proportion of people develop severe primary tumour-related complications [31, 32], it has been suggested that palliative resection does not prolong survival [33]. In our study, surgical resection was proved beneficial for survival. For CRC patients with lung metastasis, the 1- and 3-year OS rates were 61.7% vs. 41.7 and 25.7% vs. 9.7%, respectively. For those with and without primary tumour resection, the rates were 79.4% vs. 55.3 and 50.1% vs. 17.9%, respectively, among patients with lung metastasis only. In addition, evidence from population-based cancer survival analyses and clinical trial reviews suggests that chemotherapy improves survival in patients with CRC, particularly in stage IV patients [34]; similarly, in the current study, chemotherapy was shown to be beneficial to OS in both patient groups (either patients with lung metastasis from CRC or patients with lung metastasis only). The results also revealed that chemotherapy reduced the risk of cancer-specific death in patients with lung metastasis from colorectal cancer (Supplemental Fig. 1–1), and chemotherapy was also effective in reducing the risk of noncancer-specific death in this population (Table 3).

The data for 70 patients with colorectal cancer lung metastasis in our hospital were evaluated. The incidence of lung metastasis 1 year after primary resection was 40.7%, and the probability of lung metastasis after 2 years was 20.0%, which suggests that we should identify lung metastasis as soon as possible, especially for patients with high-risk factors, and regular CT reviews should be conducted. In comparison with a previous study, Facciorusso A. et al. [35]. defined the features of advanced colorectal adenomas associated with recurrence and classified the population according to their clinical characteristics in terms of the risk of recurrence to assist doctors in generating more individualized surveillance recommendations. In our study, we identified that age, race, grade, T stage, N stage, metastasis to other organs (including the liver, brain and/or bone), insurance status, and site of tumour growth were associated with the risk of lung metastasis from CRC. However, there are no risk classes to guide the clinical identification of specific high-risk patients among CRC patients, so further research is warranted in the future.

Our study has clear limitations. First, the manuscript lacks of granularity in the SEER database as it relates to the therapy sequencing and timing, patient comorbidities, and timing of clinical progression. Numerous data were missing in the SEER registry. Second, the differences in race, treatment methods and some baseline data could lead to bias when using data from 70 patients in China to verify the nomogram established from the SEER database in the US. Third, in addition to clinical baseline data, many biomarkers are associated with prognosis in CRC patients, such as RAS, BRAF, and MMR/MSI [36–38]. Several inflammatory biomarkers have been evaluated in patients with metastatic colorectal cancer. There is an article suggesting that the lymphocyte to monocyte ratio can be used to predict survival of colorectal liver metastasis [39]. In future research, more emphasis will be placed on conducting a prospective multicentre research project for enrolling large sample cases and complementing data related to molecular markers and inflammatory biomarkers to explore independent prognostic factors affecting Chinese patients with colorectal cancer lung metastasis.

## Conclusion

In summary, we developed two nomograms to predict OS in CRC patients with lung metastasis and lung metastasis only. It is recommended to use nomograms as a useful tool to predict prognosis at different time points in CRC patients with lung metastasis and to help clinicians choose appropriate treatment options for different populations. In addition, advanced age, nonwhite race, female sex, poor differentiation, T4 stage, lymph node metastasis, liver metastasis, brain metastasis, bone metastasis, being unmarried, and rectal cancer are associated with an increased risk of lung metastasis.

## Supplementary Information

**Additional file 1: Supplemental Table 1.** Clinical and demographic characteristics of the external cohort.

**Additional file 2: Supplemental Table 2.** Univariate and multivariate analysis for the presence of lung metastasis in CRC patients.

**Additional file 3: Supplemental Table 3.** Clinical and pathological characteristics, treatment modalities, and outcomes.

**Additional file 4: Supplemental Figure 1.** 1 Competing risk analyses for CRC patients with lung metastasis in training cohort according to (A) Grade, (B) Age, (C) Race, (D) Site, (E) Bone metastasis, (F) Brain metastasis, (G) Insurance status, (H) Chemotherapy. 2 Competing risk analyses for CRC patients with lung metastasis in training cohort according to (I) Liver metastasis, (J) Marital status, (K) N stage, (L) Radiotherapy, (M) Gender, (N) Surgery, (O) T stage.

**Additional file 5: Supplemental Figure 2.** Calibration curve of the nomogram predicting 1- and 3-year OS rates of CRC patients with lung metastasis in the external validation cohort (A) Calibration curve of the nomogram predicting 1- and 3-year OS rates of CRC patients with lung-only metastasis in the external validation cohort (B).

## Data Availability

Data files from SEER database were downloaded directly from the SEER website, https://seer.cancer.gov/. The data from Shanghai East hostipal that support the fndings of this study are available from the corresponding author upon reasonable request.

## Abbreviations

CRC: Colorectal cancer
SEER: Surveillance, Epidemiology and End Results
OS: Overall survival
CSS: Cancer-specific survival
NCSS: Non-cancer-specific survival
CSD: Cancer-specific death
AJCC/UICC: American Joint Committee on Cancer/International Union Against Cancer
TNM: Tumour–node–metastasis
C-index: Concordance index
