# Supplementary material for: Survival nomograms for colorectal carcinoma patients with lung metastasis and lung-only metastasis, based on the SEER database and a single-center external validation cohort
Source: BMC Gastroenterol. 2022 Nov 5;22:446. doi: 10.1186/s12876-022-02547-9 (PMC9636633; doi:10.1186/s12876-022-02547-9)
Supplement: Supplementary file 1 — Additional file 1: Supplemental Table 1. Clinical and demographic characteristics of the external cohort. [file 12876_2022_2547_MOESM1_ESM.docx]

**Supplemental Table 1 Clinical and demographic characteristics of the external cohort**

| Characteristic | Patients with lung metastasis (N=2365) | Patients with lung-only metastasis (N=565) |
| --- | --- | --- |
| **Age** |  |  |
| <65 | 1271(53.7) | 288(51.0） |
| ≥65 | 1021(43.2) | 277(49.0) |
| **Race** |  |  |
| White | 1700(71.9) | 406(71.9) |
| Black | 385(16.3) | 73(12.9) |
| Others | 280(11.8) | 86(15.1) |
| **Gender** |  |  |
| Female | 1071(45.3) | 287(50.8） |
| Male | 1294(54.7) | 278(49.2) |
| **Grade** |  |  |
| Grade I | 134(5.7) | 50(5.3) |
| Grade II | 1230(52.0) | 314(55.6) |
| Grade III | 274(11.6) | 67(11.9) |
| Grade IV | 37(1.6) | 14(2.5) |
| Unknown | 690(29.2) | 140(24.8) |
| **AJCC T stage** |  |  |
| T1 | 100(4.2) | 15(2.7) |
| T2 | 45(1.9) | 21(3.7) |
| T3 | 508(21.5) | 176(31.2) |
| T4 | 1606(23.0) | 152(26.9) |
| TX | 1216(51.4) | 201(35.6) |
| **AJCC N stage** |  |  |
| N0 | 790(33.4) | 192(34.0) |
| N1 | 737(31.2) | 187(33.1) |
| N2 | 450(19.0) | 120(21.2) |
| N3 | 288(16.4) | 66(11.7) |
| **Chemotherapy** |  |  |
| Yes | 4700(67.4) | 412(72.9) |
| No/Unknown | 2277(32.6) | 153(27.1) |
| **Site** |  |  |
| Right-sided colon | 699(31.1) | 146(25.8) |
| Left-sided colon | 637(26.6) | 134(23.7) |
| Rectum | 764(32.3) | 225(39.8) |
| Rectosigmoid | 265(11.2) | 60(10.6) |
| **Surgery** |  |  |
| No | 2008(84.9) | 435(77.0) |
| Yes | 333(14.1) | 126(22.3) |
| Unknown | 24(1.0) | 4(0.7) |
| **Osseous metastasis** |  | - |
| No | 2094(88.5) |  |
| Yes | 218(9.2) |  |
| Unknown | 53(2.2) |  |
| **Brain metastasis** |  | - |
| No | 2242(94.8) |  |
| Yes | 64(2.7) |  |
| Unknown | 59(2.5) |  |
| **Liver metastasis** |  | - |
| No | 623(26.3) |  |
| Yes | 1725(72.9) |  |
| Unknown | 17(0.7) |  |
