# Supplementary material for: Survival nomograms for colorectal carcinoma patients with lung metastasis and lung-only metastasis, based on the SEER database and a single-center external validation cohort
Source: BMC Gastroenterol. 2022 Nov 5;22:446. doi: 10.1186/s12876-022-02547-9 (PMC9636633; doi:10.1186/s12876-022-02547-9)
Supplement: Supplementary file 2 — Additional file 2: Supplemental Table 2. Univariate and multivariate analysis for the presence of lung metastasis in CRC patients. [file 12876_2022_2547_MOESM2_ESM.docx]

**Supplement Table2 Univariate and multivariate analysis for the presence of lung metastasis in CRC patients**

| Characteristic | No. of CRC patients | | Univariable | | Multivariable | |
| --- | --- | --- | --- | --- | --- | --- |
|  | Patients with LM  (N=6977) | Patients without LM  (N=122166) | χ2 | *p* value | OR (95% CI) | *p* value |
| **Age** |  |  | 61.75 | <0.001 |  | <0.001 |
| <65 | 3724(53.4) | 59300(48.5) |  |  | 1 |  |
| ≥65 | 3253(46.6) | 62866(51.5) |  |  | 1.204(1.116-1.299) |  |
| **Race** |  |  | 108.3 | <0.001 |  | <0.001 |
| White | 5124(73.4) | 95664(78.3) |  |  | 1 |  |
| Black | 1101(15.8) | 14549(11.9) |  |  | 1.240(1.114-1.380) |  |
| Others | 752(10.8) | 11953(9.8) |  |  | 1.249(1.105-1.411) |  |
| **Gender** |  |  | 19.74 | <0.001 |  | <0.001 |
| Female | 3146(45.1) | 58423(47.8) |  |  | 1 |  |
| Male | 3831(54.9) | 63743(52.2) |  |  | 0.905(0.841-0.974) |  |
| **Grade** |  |  | 2369 | <0.001 |  | <0.001 |
| Grade I | 313(4.5) | 9855(8.1) |  |  | 1 |  |
| Grade II | 3715(53.2) | 80811(66.1) |  |  | 1.144(0.962-1.361) |  |
| Grade III | 1034(14.8) | 18095(14.8) |  |  | 0.967(0.798-1.173) |  |
| Grade IV | 171(2.5) | 3508(2.9) |  |  | 0.871(0.663-1.144) |  |
| Unknown | 1744(25.0) | 9897(8.1) |  |  | 1.636(1.357-1.972) |  |
| **AJCC T stage** |  |  | 12101 | <0.001 |  | <0.001 |
| T0 | 9(0.1) | 47(0.0) |  |  | 1 |  |
| T1 | 904(13.0) | 22043(18.0) |  |  | 0.827(0.265-2.583) |  |
| T2 | 130(1.9) | 16043(13.2) |  |  | 0.25(0.079-0.797) |  |
| T3 | 2028(29.0) | 60033(49.1) |  |  | 0.573(0.184-1.786) |  |
| T4 | 1606(23.0) | 19415(15.9) |  |  | 0.933(0.299-2.912) |  |
| T_X_ | 2300(33.0) | 4585(3.8) |  |  | 1.731(0.555-5.395) |  |
| **AJCC N stage** |  |  | 5942 | <0.001 |  | <0.001 |
| N0 | 2317(33.2) | 69639(57.0) |  |  | 1 |  |
| N1 | 2369(34.0) | 32660(26.7) |  |  | 1.233(1.201-1.332) |  |
| N2 | 1293(18.5) | 18095(14.8) |  |  | 1.569(1.401-1.757) |  |
| N3 | 998(14.3) | 1772(1.5) |  |  | 1.559(1.352-1.797) |  |
| **Bone metastasis** |  |  | 4714 | <0.001 |  | <0.001 |
| No | 6107(87.5) | 121180(99.2) |  |  | 1 |  |
| Yes | 672(9.7) | 839(0.7) |  |  | 3.689(3.156-4.312) |  |
| Unknown | 198(2.8) | 147(0.1) |  |  | - |  |
| **Brain metastasis** |  |  | 1511 | <0.001 |  | <0.001 |
| No | 6564(94.0) | 121804(99.8) |  |  | 1 |  |
| Yes | 185(2.7) | 182(0.1) |  |  | 8.337(5.915-11.750) |  |
| Unknown | 228(3.3) | 180(0.1) |  |  | - |  |
| **Liver metastasis** |  |  | 17419 | <0.001 |  | <0.001 |
| No | 1892(27.2) | 106471(87.1) |  |  | 1 |  |
| Yes | 5019(71.9) | 15622(12.8) |  |  | 10.888(10.01-11.84) |  |
| Unknown | 66(0.9) | 73(0.1) |  |  | - |  |
| **Marital status** |  |  | 77.40 | <0.001 |  | 0.004 |
| Married | 3299(47.3) | 64205(52.5) |  |  | 1 |  |
| Single | 3203(45.9) | 49823(40.8) |  |  | 1.114(1.035-1.200) |  |
| Other | 475(6.8) | 8138(6.7) |  |  | - |  |
| **Insurance status** |  |  | 135.6 | <0.001 |  | - |
| Insured | 4002(57.4) | 80165(65.6) |  |  | 1 |  |
| Uninsured | 401(5.7) | 4292(3.5) |  |  | 1.318(1.154-1.506) |  |
| Unknown | 2574(36.9) | 37709(30.9) |  |  | - |  |
| **Site** |  |  | 479.4 | <0.001 |  | <0.001 |
| Right-sided colon | 2168(31.1) | 50693(41.5) |  |  | 1 |  |
| Left-sided colon | 1854(26.6) | 34231(28.0) |  |  | 1.214(1.105-1.333) |  |
| Rectum | 2165(31.0) | 27066(22.2) |  |  | 2.096(1.906-2.304) |  |
| Rectosigmoid | 790(11.3) | 10176(8.3) |  |  | 1.654(1.456-1.879) |  |
| LM, lung metastasis; CRC, colorectal cancer. | | | | | | |
