# Supplementary material for: Survival nomograms for colorectal carcinoma patients with lung metastasis and lung-only metastasis, based on the SEER database and a single-center external validation cohort
Source: BMC Gastroenterol. 2022 Nov 5;22:446. doi: 10.1186/s12876-022-02547-9 (PMC9636633; doi:10.1186/s12876-022-02547-9)
Supplement: Supplementary file 3 — Additional file 3: Supplemental Table 3. Clinical and pathological characteristics, treatment modalities, and outcomes. [file 12876_2022_2547_MOESM3_ESM.docx]

| **Supplement Table3 Clinical and pathological characteristics, treatment modalities, and outcomes** | | | | | | | | | | | | | | |
| --- | --- | --- | --- | --- | --- | --- | --- | --- | --- | --- | --- | --- | --- | --- |
| **ID** | **Sex** | **Age** | **Site** | **T** | **N** | **CEA(ug/L)** | **Survival months** | **Organs metastasis** | **LN**  **Involvement** | **Classification of lung metastasis** | **Interval between operation and lung metastasis**  **(months)** | **Treatment after lung metastasis** | **Interval between lung metastasis and death or last follow-up (months)** | **Status** |
| 1 | F | 36 | C | T4 | N2 | 2 | 38 | - | 4 | Multiple | 9 | C | 29 | Alive |
| 2 | F | 58 | R | T4 | N2 | 22 | 16 | Liver | 4 | - | 14 | R | - | **-** |
| 3 | F | 39 | R | T3 | N2 | 15 | 29 | - | 13 | Multiple | 7 | C | 22 | Alive |
| 4 | F | 69 | C | T2 | N2 | 3 | 44 | Liver | 4 | Multiple | 31 | C R | 13 | Alive |
| 5 | M | 61 | C | T2 | N0 | 51 | 62 | Liver | - | Multiple | 50 | C R | 32 | Alive |
| 6 | M | 44 | R | T3 | N2 | 1 |  | Bone | 12 | **-** | 16 | C R | **-** | **-** |
| 7 | M | 69 | C | T4 | N0 | 33 | 35 | - | - | Multiple | 12 | R | 34 | Alive |
| 8 | F | 62 | R | Tx | Nx | 24 | 70 | Liver | 0 | Oligometastasis | 39 | C | 31 | Alive |
| 9 | M | 62 | R | T4 | N1 | 23 | 36 | - | 2 | Multiple | 12 | C R | 28 | Alive |
| 10 | M | 69 | R | T3 | N2 | 6 | 71 | Liver | 5 | Oligometastasis | 13 | S | 60 | Alive |
| 11 | M | 64 | C | T4 | N1 | 8 | 67 | - | 2 | Multiple | 36 | C R | 32 | Alive |
| 12 | M | 62 | C | T4 | N0 | 132 | 72 | - | - | Multiple | 62 | C R | 11 | Death |
| 13 | F | 56 | R | T4 | N2 | 33 | 15 | Liver Bone | 6 | Multiple | 12 | C R | 7 | Death |
| 14 | F | 42 | C | T3 | N0 | 10 | 54 | Liver | - | **-** | 35 | R | 8 | Alive |
| 15 | M | 69 | C | Tx | N0 | 4 | 50 | Liver Seminal | - | **-** | 36 | R | **-** | - |
| 16 | M | 48 | R | T4 | N0 | 4 | 34 | - | 2 | Multiple | 25 | C R | 23 | Alive |
| 17 | F | 29 | R | T3 | N0 | 2 | 32 | Liver Brain | - | Multiple | 12 | C R | 28 | Alive |
| 18 | F | 45 | R | T2 | N2 | 21 | 12 | Liver Psoas | 7 | **-** | 6 | C | **-** | - |
| 19 | F | 36 | R | T2 | N0 | 1 | 51 | - | - | Oligometastasis | 9 | R | 34 | Alive |
| 20 | M | 57 | R | T3 | N0 | 1 | 65 | Bone | - | Multiple | 24 | C R | 46 | Alive |
| 21 | F | 50 | R | Tx | Nx | 2 | 79 | Pelvic cavity | - | Multiple | 106 | C R | 44 | Alive |
| 22 | M | 32 | C | T4 | N1 | 22 | 63 | Liver Pelvic  cavity | 3 | **-** | 22 | C R | **-** | - |
| 23 | F | 45 | C | T3 | N2 | 6 | 36 | Liver Pelvic  cavity | 12 | Oligometastasis | 6 | C | **-** | - |
| 24 | F | 48 | C | T3 | N2 | 37 | 36 | Liver | 12 | Oligometastasis | 2 | C R | 36 | Alive |
| 25 | M | 55 | R | T4 | Nx | 3 | 75 | Bone | 1 | Oligometastasis | 51 | C R | 24 | Alive |
| 26 | M | 78 | C | T3 | N1 | 220 | 45 | Bone | 2 | Oligometastasis | 11 | C R | 32 | Alive |
| 27 | F | 63 | C | T4 | N0 | 10 | 43 | Liver | - | Multiple | 14 | S C R | 43 | Death |
| 28 | F | 85 | C | T3 | N1 | 6 | 35 | Liver | 2 | - | 13 | R | 22 | Death |
| 29 | F | 28 | R | T3 | N2 | 31 | 35 | Liver Bone Brain | 4 | Multiple | 10 | C | 29 | Alive |
| 30 | F | 39 | C | T3 | N0 | 33 | 31 | Liver Brain | - | Multiple | 13 | S C R | 31 | Death |
| 31 | F | 57 | R | T4 | N0 | 8 | 78 | - | - | Oligometastasis | 27 | S R | 45 | Alive |
| 32 | M | 63 | R | T4 | N2 | 38 | 62 | Liver | 7 | Multiple | 20 | C | 40 | Alive |
| 33 | F | 72 | C | T4 | N1 | 84 | 17 | - | 2 | Multiple | 7 | C | 10 | Alive |
| 34 | F | 69 | C | T2 | N1 | 67 | 35 | Bone | - | - | 26 | R | 3 | Death |
| 35 | M | 75 | R | T3 | N1 | 39 | 63 | Liver Adrenal  gland Bone | 3 | Oligometastasis | 15 | S C | 14 | Alive |
| 36 | M | 37 | C | T3 | Nx | 4 | 29 | Liver | 2 | Multiple | 7 | C R | 29 | Alive |
| 37 | F | 70 | R | Tx | Nx | 198 | 39 | Liver | - | Oligometastasis | 13 | C R | 39 | Alive |
| 38 | F | 43 | C | T3 | N2 | 205 | 36 | Liver Adrenal  gland | 6 | - | 0 | C | 36 | Alive |
| 39 | M | 68 | C | Tx | Nx | 6 | - | Bone | - | Multiple | 26 | R | - | Death |
| 40 | M | 64 | R | T3 | N01 | 11 | 40 | Liver Omentum | 3 | Multiple | 11 | C | 17 | Alive |
| 41 | F | 59 | C | T4 | N0 | 36 | 37 | - | 0 | Oligometastasis | 24 | S C R | 13 | Alive |
| 42 | M | 34 | C | T4 | N2 | 112 | 34 | Liver | 5 | Multiple | 22 | C | 12 | Alive |
| 43 | M | 55 | R | Tx | N1 | 49 | 36 | Liver | 0 | Multiple | 36 | C | 0 | Death |
| 44 | M | 37 | C | T4 | N2 | 2 | 38 | - | 4 | Multiple | 9 | C | 29 | Alive |
| 45 | M | 58 | R | T4 | N2 | 22 | 16 | Liver Spleen  Bone | 4 | - | 14 | R | - | - |
| 46 | M | 39 | R | T3 | N2 | 15 | 29 | - | 13 | Multiple | 7 | C | 22 | Alive |
| 47 | F | 72 | C | T2 | N2 | 3 | 44 | Liver | 4 | Multiple | 19 | C R | 13 | Alive |
| 48 | F | 62 | C | T2 | N0 | 51 | 62 | Liver | - | - | 24 | C R | 32 | Alive |
| 49 | F | 44 | R | T3 | N2 | 1 |  | Bone Brain  Pancreas | 12 | **-** | 17 | C R | - | - |
| 50 | M | 69 | C | T4 | N0 | 33 | 35 | - | - | Multiple | 12 | R | 34 | Alive |
| 51 | F | 62 | R | Tx | Nx | 24 | 70 | Liver | 0 | Oligometastasis | 19 | C | 31 | Alive |
| 52 | F | 64 | R | T4 | N1 | 23 | 36 | - | 2 | Multiple | 12 | C R | 28 | Alive |
| 53 | M | 69 | R | T3 | N2 | 6 | 71 | Liver | 3 | Oligometastasis | 12 | S | 60 | Alive |
| 54 | M | 65 | C | T4 | N1 | 8 | 67 | - | 5 | Multiple | 23 | C R | 32 | Alive |
| 55 | F | 64 | C | T4 | N0 | 132 | 72 | - | - | - | 15 | C R | 11 | Death |
| 56 | M | 57 | R | T4 | N2 | 33 | 15 | Liver Bone Brain | 5 | Oligometastasis | 11 | C R | 7 | Death |
| 57 | M | 56 | R | Tx | N1 | 49 | 36 | Liver | 0 | Multiple | 11 | C | 0 | Death |
| 58 | M | 39 | C | T4 | N0 | 132 | 72 | - | - | - | 12 | C | 29 | Alive |
| 59 | M | 57 | C | T4 | N2 | 2 | 38 | - | 4 | Multiple | 7 | C | 22 | Alive |
| 60 | M | 40 | C | T2 | N2 | 3 | 44 | Liver | 4 | Multiple | 9 | C R | 32 | Alive |
| 61 | F | 70 | C | T2 | N0 | 51 | 62 | Liver | - | - | 19 | C R | - | - |
| 62 | F | 65 | R | T4 | N2 | 33 | 15 | Liver Bone Brain | 5 | Oligometastasis | 23 | C R | 28 | Alive |
| 63 | F | 41 | R | Tx | Nx | 24 | 70 | Liver | 0 | Oligometastasis | 24 | C | 31 | Alive |
| 64 | M | 71 | R | T4 | N2 | 22 | 16 | Liver Spleen | 4 | - | 15 | C R | 13 | Alive |
| 65 | F | 62 | R | T3 | N2 | 15 | 29 | - | 13 | Multiple | 18 | R | 34 | Alive |
| 66 | F | 63 | C | T4 | N0 | 33 | 35 | - | - | Multiple | 17 | S | 60 | Alive |
| 67 | M | 70 | R | T3 | N2 | 6 | 71 | Liver | 3 | Oligometastasis | 19 | C R | 32 | Alive |
| 68 | M | 66 | C | T4 | N1 | 8 | 67 | - | 5 | Multiple | 12 | C R | 11 | Death |
| 69 | F | 61 | R | T4 | N1 | 23 | 36 | - | 2 | Multiple | 13 | C R | 7 | Death |
| 70 | M | 57 | R | T3 | N2 | 1 |  | Bone Brain | 12 | - | 14 | R | - | - |
| F, Female; M, Male; LN, Lymph node; -, not available; C, Chemotherapy; R, Radiotherapy; S, Surgery. | | | | | | | | | | | |  |  |  |
