# Supplementary figures and images for: Survival nomograms for colorectal carcinoma patients with lung metastasis and lung-only metastasis, based on the SEER database and a single-center external validation cohort
Source: BMC Gastroenterol. 2022 Nov 5;22:446. doi: 10.1186/s12876-022-02547-9 (PMC9636633; doi:10.1186/s12876-022-02547-9)

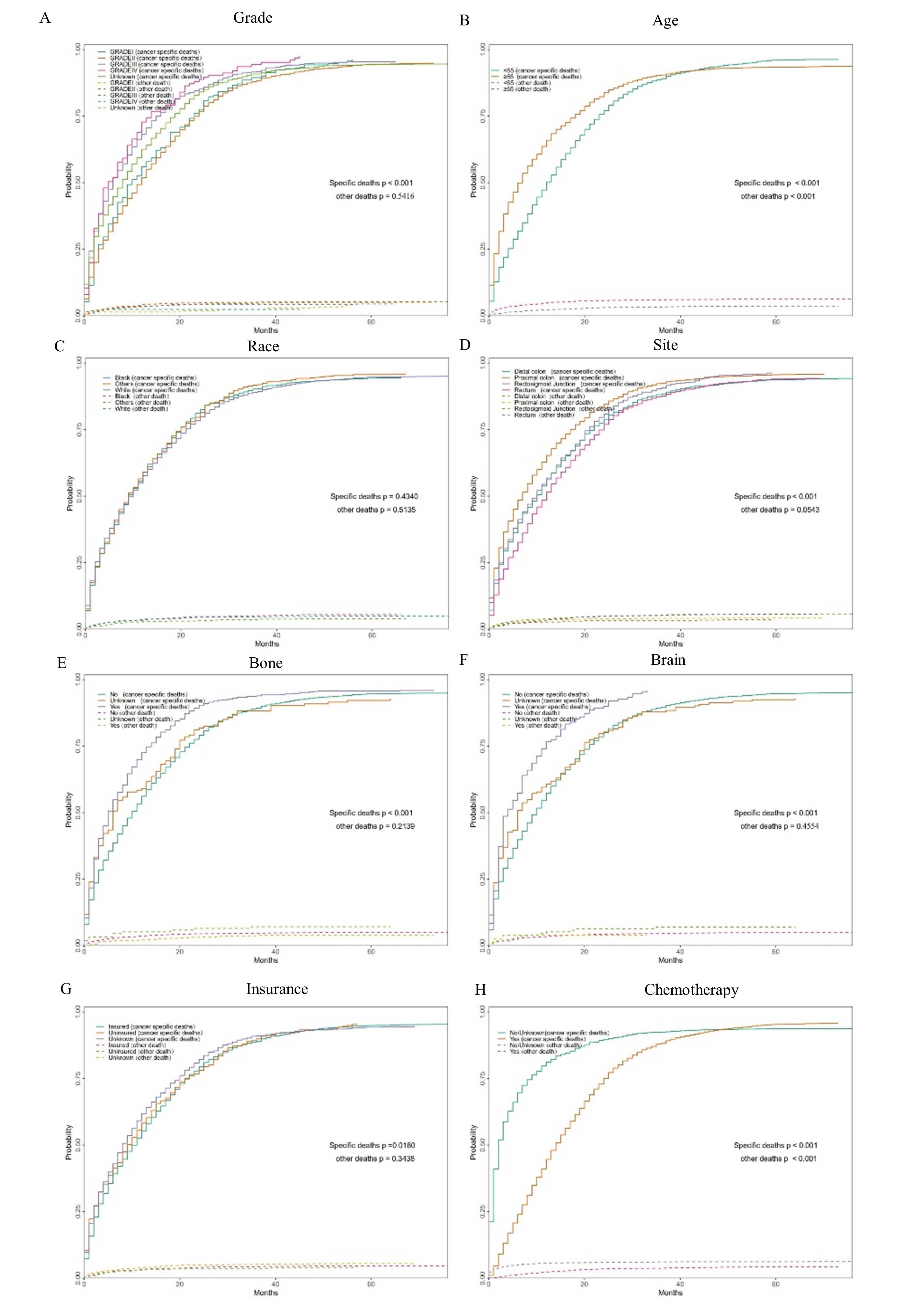

Supplement: Supplementary file 4 — Additional file 4: Supplemental Figure 1. 1 Competing risk analyses for CRC patients with lung metastasis in training cohort according to (A) Grade, (B) Age, (C) Race, (D) Site, (E) Bone metastasis, (F) Brain metastasis, (G) Insurance status, (H) Chemotherapy. 2 Competing risk analyses for CRC patients with lung metastasis in training cohort according to (I) Liver metastasis, (J) Marital status, (K) N stage, (L) Radiotherapy, (M) Gender, (N) Surgery, (O) T stage. [file 12876_2022_2547_MOESM4_ESM.zip › Supplement Figure1-1.jpg]

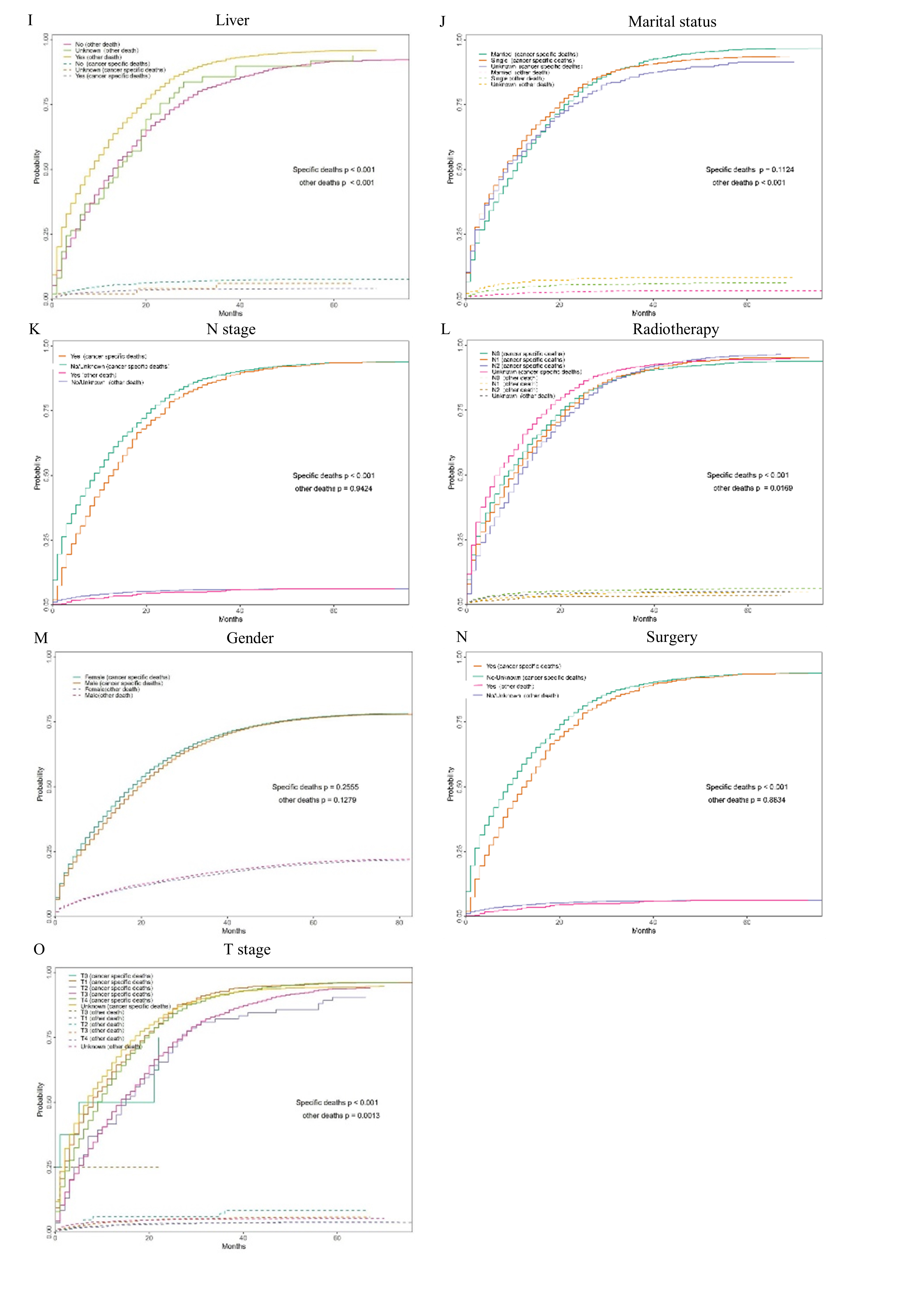

Supplement: Supplementary file 4 — Additional file 4: Supplemental Figure 1. 1 Competing risk analyses for CRC patients with lung metastasis in training cohort according to (A) Grade, (B) Age, (C) Race, (D) Site, (E) Bone metastasis, (F) Brain metastasis, (G) Insurance status, (H) Chemotherapy. 2 Competing risk analyses for CRC patients with lung metastasis in training cohort according to (I) Liver metastasis, (J) Marital status, (K) N stage, (L) Radiotherapy, (M) Gender, (N) Surgery, (O) T stage. [file 12876_2022_2547_MOESM4_ESM.zip › Supplement Figure1-2.jpeg]
